# The Effect of Temozolomide/Poly(lactide-*co*-glycolide) (PLGA)/Nano-Hydroxyapatite Microspheres on Glioma U87 Cells Behavior

**DOI:** 10.3390/ijms13011109

**Published:** 2012-01-19

**Authors:** Dongyong Zhang, Ang Tian, Xiangxin Xue, Mei Wang, Bo Qiu, Anhua Wu

**Affiliations:** 1 Departments of Neurosurgery, The First Affiliated Hospital of China Medical University, 155 Nanjingbei Street, Heping District, Shenyang 110001, China; E-Mails: dongyong0622@hotmail.com (D.Z.); fhxue2002@sohu.com (B.Q.); 2 School of Materials and Metallurgy, Northeastern University, No.11, Lane 3, WenHua Road, HePing District, Shenyang 110819, China; E-Mails: Ibrahimovic1002@126.com (A.T.); wmei1010@tom.com (M.W.)

**Keywords:** controlled release, glioma, hydroxyapatite, Poly (lactide-*co*-glycolide) PLGA, temozolomide (TMZ)

## Abstract

In this study, we investigated the effects of temozolomide (TMZ)/Poly (lactide-*co*-glycolide)(PLGA)/nano-hydroxyapatite microspheres on the behavior of U87 glioma cells. The microspheres were fabricated by the “Solid/Water/Oil” method, and they were characterized by using X-Ray diffraction, scanning electron microscopy and differential scanning calorimetry. The proliferation, apoptosis and invasion of glioma cells were evaluated by MTT, flow cytometry assay and Transwell assay. The presence of the key invasive gene, α_V_β3 integrin, was detected by the RT-PCR and Western blot method. It was found that the temozolomide/PLGA/nano-hydroxyapatite microspheres have a significantly diminished initial burst of drug release, compared to the TMZ laden PLGA microspheres. Our results suggest they can significantly inhibit the proliferation and invasion of glioma cells, and induce their apoptosis. Additionally, α_V_β3 integrin was also reduced by the microspheres. These data suggest that by inhibiting the biological behavior of glioma cells *in vitro*, the newly designed temozolomide/PLGA/nano-hydroxyapatite microspheres, as controlled drug release carriers, have promising potential in treating glioma.

## 1. Introduction

Glioblastoma, the most common type of primary brain tumor, is considered to be one of the most severe forms of human cancer. Despite advances in early diagnosis and therapeutic strategies, the two-year survival rate is 26.5 percent in patients with such a condition [1]. In treating glioblastoma, temozolomide (TMZ), an oral alkylating chemotherapeutic drug has often been used as it readily crosses the blood–brain-barrier (BBB) [2]. Unfortunately, because of the short serum half-life and dose-limiting side effects [3], systemic delivery of TMZ only produces modest benefit as a supplement to radiotherapy [1,4]. Recently, a variety of approaches have been employed to enhance the loading of TMZ into materials such as Poly (lactide-co-glycolide) (PLGA) microspheres [5,6].

PLGA has a long history of use as an excellent biomaterial due to its biocompatibility and natural degradability [7,8]. Previous researches reported that the continuous delivery of TMZ via the drug laden PLGA microparticles fabricated by the “Emulsifying-Solvent Evaporation” method showed excellent processability and controllable degradability, and that the cytotoxicity of TMZ to C6 glioma cell line was enhanced when TMZ was delivered from a PLGA carrier [5]. However, the degradation product of PLGA is acidic and could initiate inflammation [9,10]. Therefore, it was thought that the addition of hydroxyapatite (HA) could mitigate this problem due to the basic nature of its metabolites [11]. HA is a major component of bone, exhibiting good biocompatibility. It has been widely investigated as a drug carrier for the delivery of various pharmaceutical molecules due to its biocompatible, nontoxic, and non-inflammatory properties [12,13]. Compared with the micro-HA particles, the use of nanoscale HA (nHA) as adsorbent for molecules has been well-recognized as having excellent affinity to biological substances such as collagen, proteins, enzymes, cells, and viruses [14].

Many research groups have demonstrated that incorporation of nHA into PLGA microspheres could enhance the encapsulation efficacy (EE) of the drugs and prolong the duration of release [15–17]. However, most of these studies were limited to the evolution of the drug (or protein)/PLGA/nHA controlled release system. Few studies have discussed the influence of nHA morphology on the drug release profile. As the adsorption behavior of nHA is controlled by the morphology [18], we hypothesize that the adsorption ability of the nHA on drugs, reduced by the morphology of nHA, is critical for the EE and the controlled delivery during the degradation process.

In this study, the investigations of the response of U87 glioma cells to TMZ/PLGA/nHA microspheres were carried out in two systems: (i) TMZ laden on PLGA/spherical nHA (TMZ/PLGA/S-nHA); and (ii) TMZ laden on PLGA/rod-like nHA (TMZ/PLGA/R-nHA). The proliferation, apoptosis and invasion of the U87 glioma cell line, were evaluated and compared with those treated with TMZ and TMZ/PLGA. We further explored the mechanism of the effect of TMZ/PLGA/nHA microspheres on U87 glioma cells.

## 2. Results and Discussion

### 2.1. Characterization of TMZ/nHA Synthesis

**Figure 1:**
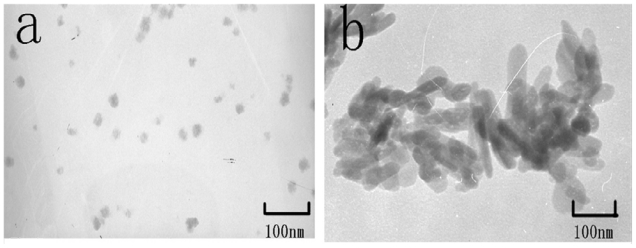
The morphology of the two types of nanoscale HA (nHA). (**a**) spherical nHA; (**b**) rod-like nHA.

Figure 1 shows the morphology of the two types of nHA prepared using the hydrothermal synthesized method and the hydrothermal homogeneous precipitation method, respectively. The results revealed that different morphologies of nHA had been prepared successfully, and the morphologies of the spherical nHA and rod-like nHA were consistent with our previous study [19]. Rod-like nHA were 80 ± 10 nm in length and 20 ± 10 nm in width, spherical nHA were 25 ± 5 nm in diameter. The drug molecules were adsorbed physically on the nHA surface. The affinity between the drug molecule and nHA mainly depends on the morphology of the nanoparticles [18], with stronger drug adhesion strength observed with the spherical nHA than with the rod-like nHA. After pre-attachment on the nHA surface, the drug content in the nHA powders laden with TMZ is shown in Table 1; the TMZ content in TMZ/R-nHA is lower than the TMZ/S-nHA.

### 2.2. The Characteristics of TMZ/PLGA/nHA Microspheres

**Figure 2:**
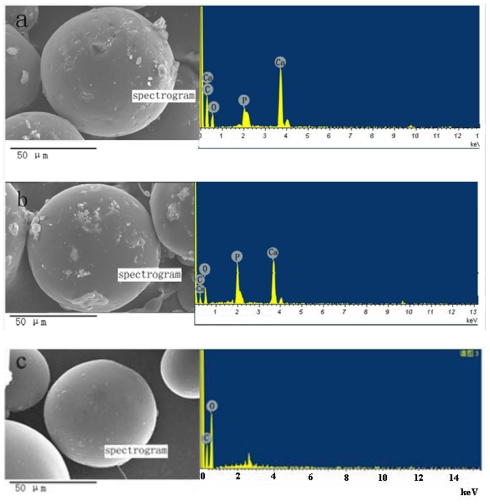
The morphology of the TMZ/PLGA/nHA and TMZ/PLGA microspheres and the energy disperse spectroscopy (EDS) patterns of wrinkling. (**a**) TMZ/PLGA/S-nHA; (**b**) TMZ/PLGA/R-nHA; (**c**) TMZ/PLGA.

**Figure 3:**
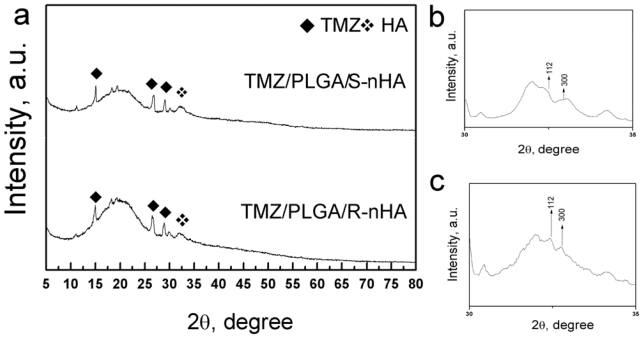
(**a**) The X-ray patterns of TMZ/PLGA/nHA microspheres; (**b**) X-ray patterns of TMZ/PLGA/S-nHA at the HA characteristic peaks; (**c**) X-ray patterns of TMZ/PLGA/R-nHA at the HA characteristic peaks.

Figure 2 showed the morphology of the TMZ/PLGA/nHA microspheres, the difference of size of the two types of microspheres was not very obvious, and the energy disperse spectroscopy (EDS) revealed that wrinkling of the surface of the microspheres was caused by the introduction of nHA. The X-ray patterns of the microspheres are shown in Figure 3. It was observed that the characteristic peak of TMZ was broadening, which indicated that the drug encapsulated in the PLGA microspheres was of low crystallinity. At the same time, the peak of nHA was also broadening caused by the nanoscale effect and low crystallinity The degree of crystallinity was calculated with the formula: $X_{C}=1-(V_{112/300}/I_{300})$. The *X_c_* is the intensity of diffraction maximum in (300), *V_112/300_* is the different value of the intensity between (112) and (300). The degree of crystallinity in the two kinds of nHA in the microspheres was 68% (rod-like) and 43% (spherical) respectively.

Table 2 reveals that the EE, microsphere size and nHA content of the two types of microspheres are similar. As the EE is related to the amount of the drug loading [5] and the amount of the TMZ/nHA between the two types of nHA powders laden with TMZ was similar, the difference of the EE was not obvious. In addition, the nHA content in the two types of microspheres is also related to the amount of TMZ/nHA. The size of microspheres was not significantly influenced by the morphology of nHA, but was mainly dependent on the other conditions of sample preparation, such as molecular masses of the polymer, polymer concentrations, and characterization techniques [5].

### 2.3. *In Vitro* Drug Release

**Figure 4:**
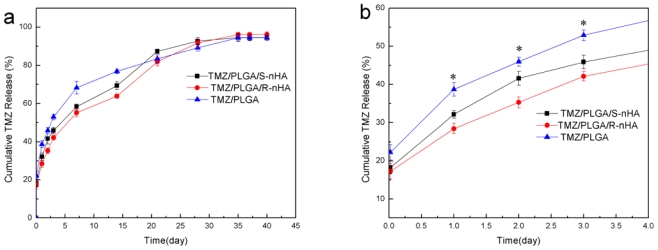
The profiles of TMZ released from TMZ/PLGA, TMZ/PLGA/S-nHA and TMZ/PLGA/R-nHA. (**a**) Within 40 days; (**b**) Within 3 days. * *p* < 0.05: significant against the TMZ/PLGA group.

**Figure 5:**
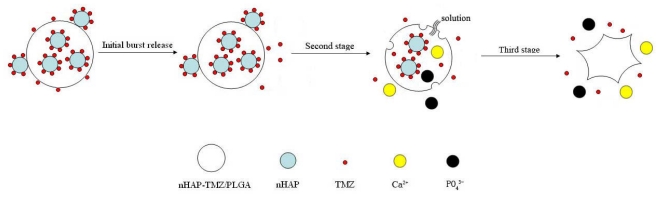
The schematic diagram of entire release process.

The release profiles of TMZ from the TMZ/PLGA/nHA microspheres are shown in Figure 4. The release of TMZ/PLGA/nHA microspheres consists of three stages. In the initial release period (within the first day), the microspheres involved with nHA, obviously reduce the initial burst from 40% to 28–31% compared with the TMZ/PLGA microspheres. As we knew the reason for the initial burst could be due to diffusional release of drug particles on the surface of microparticles [20], the initial burst of TMZ/PLGA/nHA microspheres is caused by the release of TMZ adsorbed on the microspheres surface and the TMZ/nHA attached on the microspheres (Figure 5). In our study, compared with TMZ/PLGA, the amount of drug adsorbed on the microspheres is lower caused by the pre-attachment on nHA. The reason may be that TMZ released from the microspheres was not only controlled by PLGA, but also by nHA.

In the plateau stage (from day 2 to day 14), the drug release was mainly controlled by the diffusion of the nHA attached on the surface of the microspheres. At the same time, the nHA and TMZ, encapsulated in the microspheres, will be dissolved by the acid products of PLGA, then with the nHA diffusion, the drug molecule is released into the solution through channels of the microspheres during this stage as shown in Figure 5. At the smaller scale effect and lower crystallinity of the spherical nHA, the diffusion rate of the spherical nHA was higher than for the rod-like ones both in the microspheres and aqueous phase.

In the late release stage, the release behavior was degradation-controlled. With the destruction of the microspheres, the medium penetrates into them, and the TMZ/nHA complex is released from the PLGA. The nHA dissolution into solution was accelerated with the acid degradation. Up to day 35, the cumulative release amount of the microspheres involving nHA reached about 95%.

### 2.4. Effect of TMZ/PLGA/nHA on Proliferation of Glioma Cells

**Figure 6:**
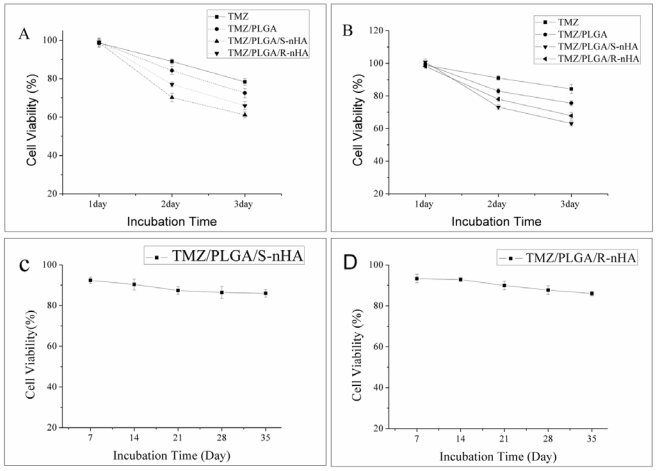
The effect of TMZ/PLGA/nHA microspheres on U87 glioma cells. The images show the cell viability of U87 glioma cells treated with TMZ, TMZ/PLGA and TMZ/PLGA/nHA, and the groups not treated with drugs served as control groups. (**A**) MTT assay for detecting the proliferation of cells; (**B**) CCK-8 assay for detecting the proliferation of cells; (**C**) The effect of release liquids (7d, 14d, 21d, 28d and 35d) from the TMZ/PLGA/S-nHA microspheres on U87 glioma cells by CCK-8 assay; (**D**) The effect of release liquids (7d, 14d, 21d, 28d and 35d) from the TMZ/PLGA/R-nHA microspheres on U87 glioma cells by CCK-8 assay.

MTT assay and CCK-8 assay were performed to investigate the effect of TMZ/PLGA/nHA on the proliferation of U87 glioma cells. The amount of TMZ that is added to the cells in each case is 50 μM. Results from our studies clearly demonstrated (shown in Figure 6A,B), on day 1, the cell viability between group TMZ, group TMZ/PLGA and groups TMZ/PLGA/nHA was not significant (*p* > 0.05). After 2 days, the cell viability of groups TMZ/PLGA/nHA and TMZ/PLGA is lower than group TMZ (*p* < 0.05). The main reason is that the serum half-life of TMZ is very short, therefore the drugs degrade very quickly in the culture medium. In the case of TMZ/PLGA, higher cell viability was shown than for groups TMZ/PLGA/nHA. Our previous studies showed the inhibitory effect of nHA on human U87 glioma cells [19], and the introduction of nHA may help to inhibit the growth of U87 glioma cells. The cell viability ratio between group TMZ/PLGA/S-nHA and group TMZ/PLGA/R-nHA was also significant (*p* < 0.05), which indicated that a different morphology of nHA in the microspheres had a different effect on the inhibition of proliferation of U87 glioma cells.

We then used the release liquids from the microspheres for longer time periods (Figure 6C,D), and found that the release liquids could also inhibit the proliferation of the U87 glioma cells. Another important point to note was that from the *in vitro* release test (Figure 4), about 45.87% (group TMZ/PLGA/S-nHA) and 42.16% (group TMZ/PLGA/R-nHA) of the TMZ was released after 3 days. This indicated that TMZ/PLGA/S-nHA, because of the high and sustained release properties, has a higher ability to inhibit the proliferation of U87 cells than TMZ/PLGA/R-nHA and TMZ. The high inhibition of proliferation of groups TMZ/PLGA/S-nHA and TMZ/PLGA/R-nHA can be sustained for a very long and its high inhibition of proliferation may go on for 35 days.

### 2.5. Effect of TMZ/PLGA/nHA on Apoptosis

Apoptosis is the process of programmed cell death that may occur in multicellular organisms, and invariably contributes to cancer cell death [21]. Therefore, we examined whether the TMZ/PLGA/nHA microspheres could induce glioma cells apoptosis.

**Figure 7:**
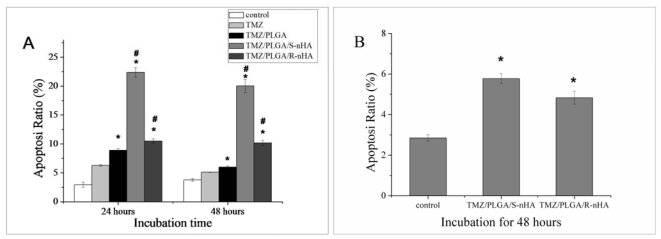
TMZ/PLGA/nHA microspheres could induce the apoptosis of U87 glioma cells. Bar graphs represent the percentage of apoptotic cells counted from each group. Data are presented as the mean of triplicate experiments. (**A**) Annexin-V/PI double-staining assay for detecting the apoptosis of U87 glioma cells. * *p* < 0.05: significant against the apoptosis ratio of TMZ group. # *p* < 0.05: significant against the apoptosis ratio of TMZ/PLGA group; (**B**) The effect of release liquids (at day 28) from the TMZ/PLGA/nHA microspheres on U87 glioma cells. * *p* < 0.05: significant against the apoptosis ratio of control group.

In our experiments, the Annexin-V/PI double-staining assay was used to detect the apoptosis of U87 glioma cells. Results of Flow Cytometry (FCM) analysis after 24 and 48 h revealed the apoptosis of U87 cells was observed when treated with TMZ, TMZ/PLGA, TMZ/PLGA/S-nHA or TMZ/PLGA/R-nHA. There was significant difference between the control group and TMZ, TMZ/PLGA, TMZ/PLGA/nHA groups (shown in Figure 7A, *p* < 0.05). Significant difference of the apoptosis ratio was also observed in the corresponding samples between groups TMZ/PLGA and TMZ/PLGA/nHA (*p* < 0.05), as well as between group TMZ/PLGA/S-nHA and group TMZ/PLGA/R-nHA(*p* < 0.01). It was shown in Figure 7A that group TMZ/PLGA/S-nHA had the highest apoptosis at 24 and 48 h, and it was almost three times higher than those of sample TMZ/PLGA and two times higher than sample TMZ/PLGA/R-nHA. FCM analysis was conducted on the release liquids from the microspheres at day 28; the results showed that TMZ released from microspheres can cause the same effect (shown in Figure 7B, *p* < 0.05). Previously the results of MTT assay indicated that groups TMZ/PLGA/nHA have higher cytotoxicity compared with group TMZ and TMZ/PLGA. Results of FCM analysis after 24 and 48 h revealed that the TMZ/PLGA/nHA microspheres have higher cytotoxicity towards U87 glioma cells than TMZ and TMZ/PLGA. Additionally, the different morphology of nHA loaded in the micropheres could have a different effect on the apoptosis of the glioma cells.

### 2.6. Invasion of U87 Glioma Cells Treated with TMZ/PLGA/nHA

One reason attributed to the poor prognosis of malignant gliomas is the highly infiltrative nature of the glioma cells. Glioma cells that migrate into the surrounding brain parenchyma escape surgical resection and other therapy. Therefore we studied the effects of TMZ/PLGA/nHA microspheres on the invasive behavior of U87 glioma cells.

**Figure 8:**
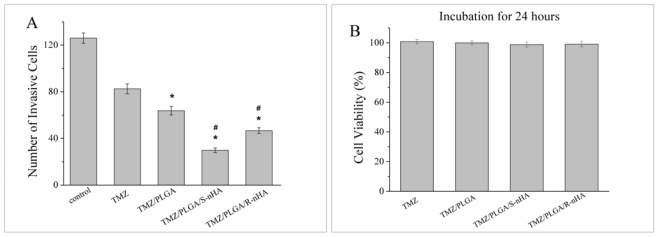
(**A**) TMZ/PLGA/nHA microspheres inhibited the invasion of U87 glioma cells. The invaded cells were stained with Giemsa. The invasion of U87 glioma cells treated with TMZ, TMZ/PLGA, TMZ/PLGA/S-nHA or TMZ/PLGA/R-nHA was reduced. * *p* < 0.05: significant against the apoptosis ratio of TMZ group. # *p* < 0.05: significant against the apoptosis ratio of TMZ/PLGA; (**B**) CCK-8 assay was used to detect the effect of TMZ, TMZ/PLGA, TMZ/PLGA/nHA microspheres on U87 glioma cells for 24 h.

We performed Transwell assays using the well characterized TMZ, TMZ/PLGA, TMZ/PLGA/S-nHA and TMZ/PLGA/R-nHA. As shown in Figure 8A, the invasion of U87 glioma cells was significantly reduced by TMZ, TMZ/PLGA, TMZ/PLGA/S-nHA and TMZ/PLGA/R-nHA, compared to that of glioma cells cultured with medium. The mean numbers of invaded cells were significantly lower in groups TMZ/PLGA/nHA than in group TMZ and TMZ/PLGA (*p* < 0.05). The decreased cell numbers seen in the Transwell assays were not due to the effect of proliferation, because the TMZ, TMZ/PLGA, TMZ/PLGA/nHA had no effect on cell viability at 24 h (Figure 8B). It was noted that, between the group TMZ/PLGA and groups TMZ/PLGA/nHA, the latter showed a low number of cells, suggesting that the nHA in TMZ/PLGA/nHA microspheres contributed to inhibition of invasion.

In summary, in the initial release period (within the first day), the TMZ/PLGA/nHA microspheres involved nHA obviously reduce the initial burst compared with TMZ/PLGA microspheres (Figure 4b). The data in Figures 6A, B, 7A and 8A suggested that at the early release period, compared with TMZ/PLGA, the TMZ/PLGA/nHA microspheres significantly inhibited the proliferation, apoptosis and invasion of U87 glioma cells, which was caused by the introduction of nHA. We then used the release liquids from the TMZ/PLGA/nHA microspheres at longer time periods (Figures 6C,D and 7B), and found that the release liquids could also inhibit the U87 glioma cells proliferation and induce apoptosis.

### 2.7. The Expression of α_V_β3 Integrin Was Reduced by TMZ/PLGA/nHA

The primary biological features of malignant glioma are high tumor cell proliferation and penetration and attachment to the normal surrounding brain tissues [22]. The attachment is mediated by cell-surface receptors known as integrins, which are crucial for cell invasion and migration [23]. Integrin is also a mediator of angiogenesis, thereby promoting tumor growth, local invasion and distant metastasis. For instance, the α_V_β3 integrin is expressed in glioma and its expression correlates with glioma grade [24]. Furthermore, α_V_β3 integrin plays an important role in promoting tumor invasion [25], and it also regulates glioma cell proliferation [26].

**Figure 9:**
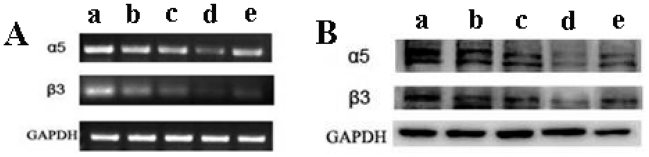
Expression of α_V_β3 integrin in U87 glioma cells. (**A**) mRNA expression of α_V_β3 integrin; (**B**) protein expression of α_V_β3 integrin. (**a**) control group (not treated with drug); (**b**) group TMZ; (**c**) group TMZ/PLGA; (**d**) group TMZ/PLGA/S-nHA; (**e**) group TMZ/PLGA/R-nHA.

In our study, we determined the effect of TMZ/PLGA/nHA microspheres on the expression of α_V_β3 integrin. As shown in Figure 9A, compared with the glioma cells treated with medium, the α_V_β3 integrin mRNAs expression was down-regulated in U87 glioma cells treated with TMZ, TMZ/PLGA and TMZ/PLGA/nHA. The α_V_β3 integrin expression in glioma cells treated with TMZ/PLGA/nHA was lower than that tested in TMZ and TMZ/PLGA glioma samples. The housekeeping gene GAPDH was used as a positive control. We then sought to confirm this finding at the protein level by performing Western blot analysis in U87 glioma cells. We found that both the α_V_ and β3 subunits were down-regulated at protein level in TMZ, TMZ/PLGA, and TMZ/PLGA/nHA treated glioma cells, and the lowest expression was in TMZ/PLGA/S-nHA treated glioma cells (Figure 9B). This indicated that the expression of α_V_β3 integrin decreases after interference by TMZ/PLGA/nHA microspheres. The α_V_β3 integrin is one of the key genes of glioma proliferation and invasion, and the microspheres may inhibit the proliferation and invasion of glioma cells through this cytokine signaling pathway.

## 3. Experimental Section

### 3.1. Materials

The starting materials employed in this study were PLGA (75:25 mole ratio of lactide to glycolide, molecule weight is 60,000 g/mol (Sigma-Aldrich, Inc., NY, USA), PVA (88% hydrolyzed, Sigma-Aldrich, Inc., NY, USA), CH_2_Cl_2_ (Shanghai Guoyao Group, Shanghai, China), dimethyl sulfoxide (DMSO, Sigma-Aldrich, Inc., NY, USA). TMZ was kindly supplied by the Tasly Pharmaceutical Co., Ltd (China). The cell-culture related reagents were Dulbecco’s modified eagle’s medium (DMEM, Hyclone, UT, USA), fetal bovine serum (FBS, Hyclone, UT, USA), MTT (Sigma-Aldrich, Inc., NY, USA), CCK-8(Wuhan Boster Bio-Engineering Limited Company, Wuhan, China), FITC Annexin V Apoptosis Detection Kit (BD Biosciences, MA, USA), Matrigel Basement membrane matrix (BD Biosciences, MA, USA), Giemsa (Sigma, St. Louis, USA), TRIzol reagent (Invitrogen, CA, USA), radioimmunoprecipitation assay (RIPA) protein extraction buffer (Applygen Technologies Inc, Beijing, China), Bradford protein assay (Applygen Technologies Inc, Beijing, China), rabbit anti-integrin α_V_ polyclonal antibody (Abcam, Cambridge, UK), rabbit anti-integrin β_3_ monoclonal antibody (Abcam, Cambridge, UK), and goat anti-rabbit horseradish peroxidase-conjugated antibody (Zhongshan Goldenbridge Biotechnology Co., LTD, Beijing, China).

### 3.2. Preparation of nHA Powder and Absorption Experiment of the TMZ/nHA

Spherical nHA was prepared by the hydrothermal synthesized method [27], and Rod-like nHA was synthesized by the hydrothermal homogeneous precipitation method [28]. The TMZ molecule pre-attached on the nHA surface was prepared using the following method; 10 mg TMZ were dissolved in 20 mL DMSO, then the DMSO was mixed with 100 mL nHA-suspended solution (100 mg/L). The suspension was kept at 4 °C under stirring for 12 h, and the deposited phase was isolated by centrifugation, washed with distilled water three times and then freeze-dried. The concentration of TMZ in TMZ/nHA was calculated as follows:

A series of known concentration of TMZ solutions (dissolved in DMSO) were analyzed by UV to determine the absorbance at λ max 327 nm. Then taking the concentration as the X-axis and the absorbance as the Y-axis, the UV curve standardization evaluation was drawn to fit the linear Equation (1).

(1) $$A=1.056\times 10^{-2}C+0.480\times 10^{-2}$$

Where the *A* is the UV-absorbance, the *C* is the content of TMZ (μM), the linear Correlation Coefficient was 0.99974.

To determine the concentration of the amount of TMZ, a known amount of nHA powder laden TMZ was dissolved in 3 mL DMSO, using a UV-spectrophotometer to determine the absorbance of the samples at λ max 327 nm. Subsequently, the content of the drug was calculated by the Equations (1) and (2).

(2) $$m(mg)=C(\mu mol/L)\times 10^{-6}\times v(mL)\times 10^{-3}\times M(g/mol)\times 10^{3}$$

Where the *m* iss the amount of the TMZ, *C* is the content of TMZ, *v* is the volume of the DMSO containing TMZ, *M* equals 194.15 g/mol.

### 3.3. Fabrication of TMZ/PLGA/nHA Microspheres by S/O/W Method

TMZ/PLGA/nHA microspheres were prepared using the S/O/W method [5]. Briefly, a known amount of TMZ/nHA was dispersed in 1.5 mL of CH_2_Cl_2_ with 200 mg PLGA by sonication at 90 W for 3 min under 4 °C. The s/o dispersion was then poured into 80 mL of 2% (w/v) PVA aqueous solution saturated with 400 mg TMZ to form S/O/W suspension. The s/o/w solution was then stirred at 600 rpm for 5 h to enable evaporation of the organic solvent. The resultant microspheres were collected by centrifugation, washed with distilled water three times and then freeze-dried. In addition, in our study, the microspheres without nHA were regarded as the control group, and the drug amount in fabricating the microspheres was defined as 10 mg. In all experimental biological cases, the same weight of TMZ was used.

### 3.4. Characterization of Microspheres

X-Ray diffraction (XRD) was used to determine the nature of the microspheres and the nHA powder laden TMZ by a Huber D8211 diffractometer with Cu Kα radiation. Transmission electron microscopy (TEM, FEI tecnai-G2) was employed for the morphology of the nHA. Scanning electron microscopy (SEM JSM-6500F) was employed to observe the TMZ/PLGA/nHA microspheres. The nHA proportion in microspheres was examined by differential scanning calorimetry (DSC) (SDT2960 Simultaneousy DSC-TGA), the samples were heated from 25 °C to 600 °C at a constant temperature increment of 10 °C/min and purged with nitrogen gas at 30 mL/min.

### 3.5. Determination of TMZ Encapsulation Efficiency in Microspheres

The drug EE in microspheres was measured after extraction from the microspheres. The microspheres (2 mg) laden TMZ/nHA were dissolved in 3 mL DMSO, and the suspension was analyzed by a UV spectrophotometer at a wave length of 327 nm with DMSO as reference. The EE was calculated with the following Equation (3)

(3) $$EE(\%)=D_{m}\times 100/D_{t}$$

Where *D_t_* was the amount of TMZ for the preparation and Dm was the amount of TMZ in the freeze-dried microspheres.

### 3.6. *In Vitro* Release of TMZ from Microspheres

*In vitro* TMZ release experiments of TMZ/PLGA/nHA microspheres were performed in a shaking incubator at 60 rpm, 37° C. The microspheres (15 mg) were re-suspended in 15 mL PBS (pH 7.4). These samples were incubated in a shaking bath. At regular intervals (0.5 h, 1, 2, 3, 7, 14, 21, 28 and 35 days), the samples were collected and centrifuged at 2000 rpm for 5 min, and then dissolved in DMSO. The amount of TMZ in the microspheres was determined by UV analysis described above. All experiments were repeated three times.

### 3.7. Cell Culture

Human glioma cell line, U87, was purchased from The Cell Bank of Type Culture Collection of Chinese Academy of Sciences and maintained in Dulbecco’s modified eagle’s medium (DMEM) with supplements of 10% FBS, 100 IU/mL penicillin G, and 100 μg/mL streptomycin. The medium was changed every other day.

### 3.8. Cell Viability

Exponentially growing U87 glioma cells were seeded in 96-well plates at a density of 6 × 10^3^ cells/well (200 μL per well) overnight. Then the medium was changed with 200 μL medium with TMZ, TMZ/PLGA, TMZ/PLGA/S-nHA and TMZ/PLGA/R-nHA. The amount of TMZ that added to the cells in each case was 50 μM. The amount of microspheres (TMZ/PLGA, TMZ/PLGA/S-nHA and TMZ/PLGA/R-nHA) used in each case is 83 μg/mL, 106 μg/mL and 55 μg/mL. The plate was incubated for 1, 2, 3 days. MTT (20 μL, 5 mg/mL) was added to each well. During the incubation time, we did not change the medium. After 4 h of incubation at 37 °C, the culture medium was removed and the intracellular formazan crystals were solubilized with 150 μL DMSO. After shaking for 10 min, the absorbance of each well at 490 nm was measured by an enzyme immunoassay instrument. All experiments were repeated in triplicate. Cell viability was determined by the following equation: cell viability (%) = (Abs test cells/Abs control cells) × 100. Cell viability was also assessed using a CCK-8 following the manufacturer’s instructions.

### 3.9. Flow Cytometry (FCM) Analysis

After 24 and 48 h in culture, U87 glioma cells, untreated or treated with TMZ, TMZ/PLGA, TMZ/PLGA/S-nHA and TMZ/PLGA/R-nHA, were analyzed by FCM to assess the apoptosis of glioma cells by FITC Annexin V Apoptosis Detection Kit. The amount of TMZ added to the cells in each case was 50 μM. Briefly, the cells were washed with Phosphate buffered saline (PBS) and then re-suspended in Binding Buffer at a concentration of 1 × 10^6^ cells/mL. To 100 μL of cells, 5 μL of FITC Annexin V and 5 μL PI were added and stained for 15 min at RT (25 °C) in the dark. Then, the cells were added with 400 μL Binding Buffer and analyzed BD FACSCalibur (BD Biosciences, MA, USA)

### 3.10. Invasion Assay

Invasion assays were performed in a 24-well Transwell chamber (6.5 mm diameter 8.0 μm pore size polycarbonaate filters, Costa, Corning, NY, USA). Matrigel Basement membrane matrix was diluted by the DMEM medium and coated in the upper Transwell chamber. Cells were seeded to coated chamber (4 × 10^3^ cells) in 200 μL of serum-free medium without or with TMZ, TMZ/PLGA, TMZ/PLGA/S-nHA and TMZ/PLGA/R-nHA. Another 500 μL of DMEM medium with 20% FBS was added in the lower parts of the chambers. After 24 h incubation, the upper Matrigel coated surface was wiped off using a cotton swab. Cells migrated through the filters were fixed, stained with Giemsa. Nuclei of invasive cells were counted in five high-power fields (200×) and the values expressed as the mean ± SE.

### 3.11. Semiquantitative Detection of α_V_β_3_ Integrin mRNA with Reverse Transcription Polymerase Chain Reaction (RT-PCR)

All samples of cells which were treated by TMZ, TMZ/PLGA, TMZ/PLGA/S-nHA and TMZ/PLGA/R-nHA for 48 h were placed on ice, from which RNA was extracted using TRIzol reagent. RNA(500 ng) was reverse transcribed into cDNA. The primer sequences of α_V_ integrin were as follows: 5′CAGCCCTACATTATCAGAGCAA3′ for the forward primer and 5′GTTCACGGCAAAGTAGTCACAG3′ for the reverse primer, giving a 299 bp amplified fragment. The primer sequences of β_3_ integrin were as follows: 5′CTCATCACCATCCACGAC3′ for the forward primer and 5′CCACATACTGACATTCTCCC3′ for the reverse primer, giving a 279 bp amplified fragment. The primer sequences of GAPDH were as follows: 5′AGGTCGGAGTCAACGGATTTG3′ for the forward primer and 5′GTGATGGCATGGACTGTGGT3′ for the reverse primer, giving a 531 bp amplified fragment. Reverse transcriptase PCR was set up using the PCR cycle [95 °C for 5 min, (95 °C for 30 s, 55–60 °C for 30 s, and 72 °C for 30 s) × 30 cycles, 72 °C for 10 min]. PCR products were resolved on a 1.6% agarose gel, visualized, and photographed under UV light.

### 3.12. Western Blot Analysis

U87 glioma cells were treated with TMZ, TMZ/PLGA, TMZ/PLGA/S-nHA and TMZ/PLGA/R-nHA for 48 h. Cells were collected and extracted with RIPA protein extraction buffer. Protein concentrations were determined by the Bradford protein assay. After overnight transfer onto nitrocellulose membranes, blots were blocked with 5% skimmed milk in 1 × TBST. Blots were then incubated overnight at 4 °C with primary antibody: rabbit anti-integrin α_V_ polyclonal antibody or rabbit anti-integrin β_3_ monoclonal antibody. The following secondary antibody detection was used: goat anti-rabbit horseradish peroxidase-conjugated antibody. Immunoreactive bands were visualized using chemiluminescence ECL Western blotting detection reagents on Hyperfilm-MP autoradiography film. GAPDH (housekeeping gene) antibody was used to verify that similar amounts of protein were loaded in all lanes.

### 3.13. Statistical Analysis

Statistics were generated for all quantitative data with the presentation of means ± SE. The significance was determined by one way analysis of variance (ANOVA) using the SPSS10.0 statistical software.

## 4. Conclusions

The morphology of the two types of nHA were prepared using the hydrothermal synthesized method and the hydrothermal homogeneous precipitation method, TMZ/PLGA/nHA microspheres were prepared using the S/O/W method. From XRD, SEM and DSC results, it appeared that TMZ trapped in the microspheres existed in an amorphous or disordered-crystalline status in the polymer matrix. The vitro drug release assay revealed that TMZ/PLGA/nHA microspheres have a much slower burst release rate of TMZ compared to TMZ/PLGA due to the nHA controllable release. Moreover, the morphology of nHA could affect in-vitro release of the microspheres. This study also showed that the use of nHA as an additive aided the release of TMZ from microspheres while it also inhibited glioma growth and invasion when tested *in vitro* with U87 glioma cells line. Ultimately, the TMZ/PLGA/nHA microspheres have shown the best performance in TMZ controlled release as well as in inhibition of cell viability. Hence, it can be concluded that the TMZ/PLGA/nHA microspheres (especially group TMZ/PLGA/S-nHA) are promising drug delivery devices for glioma therapy.

## Figures and Tables

**Table 1:** The content of temozolomide (TMZ) in the TMZ/nHA and amount of TMZ/nHA used in fabricating.

Samples	TMZ content (wt %)	Amount of TMZ/nHA used in fabricating (mg)
TMZ/R-nHA	46.4 ± 2.03	21.6
TMZ/S-nHA	54.5 ± 3.28	18.3

**Table 2:** Microsphere size distribution, TMZ encapsulation efficacy (EE) and the proportion of the nHA in microspheres.

Samples	EE (%)	Microspheres Size (μm)	Concentration of the nHA (wt %)
TMZ/PLGA/R-nHA	84.19 ± 1.48	62.7	4.49
TMZ/PLGA/S-nHA	82.47 ± 1.9	63.9	4.39
